# Assessment of a Hotel-Based Protective Housing Program for Incidence of SARS-CoV-2 Infection and Management of Chronic Illness Among Persons Experiencing Homelessness

**DOI:** 10.1001/jamanetworkopen.2021.38464

**Published:** 2021-12-13

**Authors:** Thomas D. Huggett, Elizabeth L. Tung, Megan Cunningham, Isaac Ghinai, Heather L. Duncan, Maura E. McCauley, Wayne M. Detmer

**Affiliations:** 1 Lawndale Christian Health Center, Chicago, Illinois; 2 Section of General Internal Medicine and Center for Health and the Social Sciences, University of Chicago, Chicago, Illinois; 3 Chicago Department of Public Health, Chicago, Illinois; 4 Department of Nursing, North Park University, Chicago, Illinois; 5 Chicago Department of Family and Support Services, Chicago, Illinois

## Abstract

**Importance:**

Persons experiencing homelessness (PEH) are at higher risk for SARS-CoV-2 infection and severe illness due to COVID-19 because of a limited ability to physically distance and a higher burden of underlying health conditions.

**Objective:**

To describe and assess a hotel-based protective housing intervention to reduce incidence of SARS-CoV-2 infection among PEH in Chicago, Illinois, with increased risk of severe illness due to COVID-19.

**Design, Setting, and Participants:**

This retrospective cohort study analyzed PEH who were provided protective housing in individual hotel rooms in downtown Chicago during the COVID-19 pandemic from April 2 through September 3, 2020. Participants were PEH at increased risk for severe COVID-19, defined as (1) aged at least 60 years regardless of health conditions, (2) aged at least 55 years with any underlying health condition posing increased risk, or (3) aged less than 55 years with any underlying health condition posing substantially increased risk (eg, HIV/AIDS).

**Exposures:**

Participants were housed in individual hotel rooms to reduce the risk of SARS-CoV-2 infection; on-site health care workers provided daily symptom monitoring, regular SARS-CoV-2 testing, and care for chronic health conditions. Additional on-site services included treatment of mental health and substance use disorders and social services.

**Main Outcomes and Measures:**

The main outcome measured was SARS-CoV-2 incidence, with SARS-Cov2 infection defined as a positive upper respiratory specimen using any polymerase chain reaction diagnostic assay authorized for emergency use by the Food and Drug Administration. Secondary outcomes were blood pressure control, glycemic control as measured by hemoglobin A_1c_, and housing placements at departure.

**Results:**

Of 259 participants from 16 homeless shelters in Chicago, 104 (40.2%) were aged at least 65 years, 190 (73.4%) were male, 185 (71.4%) were non-Hispanic Black, and 49 (18.9%) were non-Hispanic White. There was an observed reduction in SARS-CoV-2 incidence during the study period among the protective housing cohort (54.7 per 1000 people [95% CI, 22.4-87.1 per 1000 people]) compared with citywide rates for PEH residing in shelters (137.1 per 1000 people [95% CI, 125.1-149.1 per 1000 people]; *P* = .001). There was also an adjusted change in systolic blood pressure at a rate of −5.7 mm Hg (95% CI, −9.3 to −2.1 mm Hg) and hemoglobin A_1c_ at a rate of −1.4% (95% CI, −2.4% to −0.4%) compared with baseline. More than half of participants (51% [n = 132]) departed from the intervention to housing of some kind (eg, supportive housing).

**Conclusions and Relevance:**

This cohort study found that protective housing was associated with a reduction in SARS-CoV-2 infection among high-risk PEH during the first wave of the COVID-19 pandemic in Chicago. These findings suggest that with appropriate wraparound supports (ie, multisector services to address complex needs), such housing interventions may reduce the risk of SARS-CoV-2 infection, improve noncommunicable disease control, and provide a pathway to permanent housing.

## Key Points

**Question:**

Was a hotel-based protective housing intervention associated with reduced incidence of SARS-CoV-2 infection among persons experiencing homelessness (PEH) in Chicago, Illinois?

**Findings:**

In this cohort study of 259 PEH, a significant reduction in SARS-CoV-2 incidence was observed during the study period among PEH provided with protective housing compared with PEH in shelters citywide. Improvements in hypertension and glycemic control were also observed; 51% were successfully housed at departure.

**Meaning:**

These findings suggest that protective housing interventions may reduce SARS-CoV-2 incidence among PEH at increased risk for severe COVID-19.

## Teaser

This cohort study examines the association of a hotel-based protective housing intervention and the incidence of SARS-CoV-2 infection among persons experiencing homelessness in Chicago, Illinois.

## Introduction

Persons experiencing homelessness (PEH) are at increased risk of becoming infected with SARS-CoV-2 and developing COVID-19. Studies across the US and Europe have identified a high incidence of SARS-CoV-2 in PEH, particularly among those in congregate settings.^1,2,3,4^ In one early study conducted at a large homeless shelter in Boston,^1^ more than one-third of residents (36%) tested positive for SARS-CoV-2, the majority (88%) of whom were asymptomatic at the time of testing. Difficulty maintaining physical distance, as well as challenges in obtaining personal protective equipment, can make SARS-CoV-2 transmission more likely among PEH.

Importantly, PEH also have a higher prevalence of underlying health conditions that place them at higher risk for severe illness or death from COVID-19. The prevalence of hypertension is estimated to be 50% among PEH in the US,^5^ with multiple studies documenting poor rates of blood pressure control.^6,7,8^ Although prevalence of diabetes in PEH may be comparable with other low-income populations,^5^ the likelihood of adequate glucose control is substantially lower^9^ resulting in higher complication rates.^10^

In April 2020, a hotel-based protective housing intervention was implemented in Chicago, Illinois, through a joint effort by the Chicago Departments of Public Health (CDPH), Family and Support Services (DFSS), and Housing (DOH), in conjunction with Lawndale Christian Health Center (LCHC), a federally qualified health center with longstanding experience in providing health care to PEH. Published assessments of interventions elsewhere have focused on widespread testing to interrupt transmission of COVID-19 in congregate settings,^4,11^ or isolation and quarantine measures for PEH who tested positive.^12^ This Chicago model, in contrast, provided protective housing for PEH who were at risk for severe illness and death from COVID-19 owing to older age or underlying health conditions. We are not aware of any published assessments of similar protective housing efforts. In addition to individualized hotel rooms, on-site health care workers provided symptom monitoring, regular SARS-CoV-2 testing, and medical care for chronic health conditions. Other key services included meal delivery, treatment of mental health and substance use disorders (SUDs), as well as social services. Although hotel conversions to expand supportive housing existed before the pandemic, the COVID-19 pandemic provided an accelerated opportunity to pilot a model targeted to PEH with high-risk health conditions.

The primary aim of this study was to describe Chicago’s hotel-based protective housing intervention and assess whether the intervention was associated with any differences in the incidence of SARS-CoV-2 infection among those participating in the intervention relative to the citywide population of sheltered PEH. Secondary aims were to assess change in high-risk health conditions during the study period, including hypertension and diabetes; describe treatment for mental health conditions and SUDs; and describe housing outcomes on departure from the intervention.

## Methods

### Study Design and Setting

We conducted a retrospective cohort study of PEH who consented to participate in a hotel-based housing intervention between April 2 and September 3, 2020. All participants were offered temporary housing in individual hotel rooms leased by the City of Chicago. Chicago’s overall response to the COVID-19 crisis among PEH, implemented with support from the Chicago Homelessness and Health Response Group for Equity (CHHRGE),^13^ has been described elsewhere.^11^ Just prior to the pandemic, there were an estimated 5390 persons experiencing homelessness in Chicago.^14^ Few (10.2%) were aged at least 60 years; the majority were male (61.6%) and non-Hispanic Black (77.0%); most resided in homeless shelters (72.2%). Available data indicated high prevalence of cardiovascular and respiratory conditions, diabetes, tobacco use, physical disabilities, mental health conditions and SUDs,^11,14^ many of which are known to increase risk of severe illness from COVID-19.^15^

On April 2, 2020, the City of Chicago established an isolation facility for PEH in downtown Chicago. The facility, which opened at a time when SARS-CoV-2 testing was not widely available, was initially intended for housing people with possible COVID-19 infection while test results were pending. However, with growing evidence of asymptomatic transmission and high infection rates in shelters across Chicago and other large metropolitan areas,^2^ it became clear that isolation of symptomatic individuals was likely insufficient to interrupt transmission of SARS-CoV-2 in homeless shelters. On April 10, the hotel was repurposed as protective housing for PEH who were at increased risk of severe illness from COVID-19 and not suspected to have SARS-CoV-2 infection.

The reporting of this study follows the Strengthening the Reporting of Observational Studies in Epidemiology (STROBE) reporting guideline for cohort studies. The study intervention received a nonresearch determination as part of a public health emergency response; retrospective assessment of the intervention was approved with a waiver of informed consent from the University of Chicago institutional review board.

### Study Participants

Eligibility for protective housing included any PEH, whether residing in a shelter, in an encampment, or on the street, who met criteria for being at increased risk for severe illness due to COVID-19, defined as (1) aged at least 60 years regardless of health conditions, (2) aged at least 55 years with any underlying health condition posing increased risk,^16^ or (3) aged less than 55 years with any underlying health condition posing substantially increased risk (eg, HIV/AIDS). Underlying health conditions included hypertension, diabetes (type 1 or 2), obesity, immune compromise, and chronic respiratory conditions.^16^ Substantially increased risk was assessed on a case-by-case basis, through discussions between team physicians and medical epidemiologists.

To identify eligible PEH, CDPH/DFSS requested that congregate shelters with at least 10 people sharing a dormitory generate a provisional list of eligible residents. An experienced physician visited each shelter to confirm eligibility, identify other potentially eligible residents, and conduct in-person risk assessments for mobility accommodations, medications, and possible withdrawal from substances. Individuals gave oral consent for participation. Race and ethnicity and other sociodemographic information were self-reported.

### Protective Housing Intervention

Protective housing included a private hotel room with a bathroom and shower, Wi-Fi, TV, and meal delivery; security and laundry services were subcontracted. Medical services included universal SARS-CoV-2 polymerase chain reaction (PCR) testing on or soon after admission; daily assessments for signs and symptoms of COVID-19; access to regular medications, including treatments for opioid, alcohol, and tobacco use disorders (eg, methadone, buprenorphine, naltrexone); primary care and behavioral medicine visits; wound and foot care; access to specialist services such as on-site podiatry; and referral to an acute care facility as necessary. COVID-19 assessments included temperature checks, oxygen saturation checks, and a symptom questionnaire. Medical staff provided 24-hour coverage and included up to 9 full-time providers (ie, physicians, nurse practitioners, and physician assistants) who typically provided care in LCHC outpatient clinics. Daily pharmacy deliveries facilitated rapid medical treatment. Social services included case management and assistance with housing.

To minimize the risk of SARS-CoV-2 spread, movement into and out of the hotel was initially restricted. During the first month of operations, when details about the transmissibility of SARS-CoV-2 infection were still emerging, participants were largely confined to their own rooms. After the first month, adjustments were made so that guests could leave to complete necessary tasks (eg, picking up monthly social security checks). Smoke breaks and other socially distanced activities were instituted, such as supervised exercise breaks on the hotel rooftop, magic shows by one of the guests, walks around the block, and other activities.

### Main Outcomes

The primary outcome of interest was the incidence of SARS-CoV-2 infection. SARS-CoV-2 infection was defined as any individual with a positive upper respiratory specimen using any PCR diagnostic assay authorized for emergency use by the Food and Drug Administration. Individuals were included in the protective housing cohort if they were admitted on or after April 10, when protective housing began, or if they converted from the isolation to protective housing cohort after negative SARS-CoV-2 testing. Incidence among the protective housing cohort was compared to incidence among PEH residing in shelters citywide. A detailed summary of the methodology for estimating SARS-CoV-2 infection among sheltered PEH in Chicago is included in the eMethods in the Supplement.

Secondary outcomes included the status of known modifiable risk factors for severe illness due to COVID-19, including hypertension and diabetes control. Repeated systolic blood pressure (SBP), diastolic blood pressure (DBP) and hemoglobin A1c (HbA_1c_) measurements were extracted from the Electronic Health Record and analyzed as continuous variables. Additional outcomes related to mental health, SUD, and housing were also recorded and summarized.

### Statistical Analysis

Sociodemographic and shelter characteristics of the sample were described. Incidence among the protective housing cohort was calculated as the number of participants who were provided with protective housing and tested positive for SARS-CoV-2 divided by the total number of participants who were provided with protective housing. Incidence among sheltered PEH citywide was calculated as the number of reported SARS-CoV-2 infections divided by the number of susceptible sheltered PEH in Chicago who were not part of the protective housing intervention (see eMethods in the Supplement for more details). Incidence was additionally calculated and graphed for each month of the intervention. The Fisher exact test of independence (2-sided) was used to detect significant differences (*P* < .05) between the incidence of SARS-CoV-2 infection among those receiving protective housing and those residing in shelters citywide.

In secondary analyses of chronic illness, we used generalized estimating equations (GEE) in population-averaged longitudinal regression models to independently assess blood pressure and HbA_1c_ measurements as a function of intervention time (baseline vs pooled follow-up measurements). The same model was applied for all outcome measures, grouping observations by patient identifier and implementing robust standard errors. Each model adjusted for all theoretically relevant confounders, including sociodemographic characteristics (age, gender, race/ethnicity, and preferred language), comorbid mental health conditions and/or SUDs, shelter of origin, day at the hotel (ie, when the observation was recorded), and total days at the hotel. The day at the hotel was modeled as a time-varying covariate; all other covariates were time invariant. While GEE is a common statistical approach used in public health (ie, average effects across a specified population), we also conducted sensitivity analyses using traditional fixed effects models to assess for within subject change over time.

## Results

Of 259 PEH admitted to the hotel from 16 homeless shelters (eFigure in the Supplement), 104 (40.2%) were aged at least 65 years; 190 (73.4%) were male, 185 (71.4%) were non-Hispanic Black, and 49 (18.9%) were non-Hispanic White (Table 1). Median (IQR) duration at the hotel was 59 (18-115) days. Overall, 16 116 daily health care encounters were provided, of which 9820 (60.9%) were for individuals designated by the medical team as high-acuity.

**Table 1.** Participant Characteristics

Participant characteristics	Participants, No. (%) (N = 259)
Age, y	
<50	23 (8.9)
50-64	132 (51.0)
≥65	104 (40.2)
Gender	
Male	190 (73.4)
Female	69 (26.6)
Race and ethnicity	
Hispanic or Latino	20 (7.7)
Non-Hispanic Black	185 (71.4)
Non-Hispanic White	49 (18.9)
Other^a^	5 (1.9)
Insurance status	
Medicaid/Medicare	204 (78.8)
Uninsured	55 (21.2)
Language preference	
English	237 (91.5)
Spanish	12 (4.6)
Other	10 (3.9)
Chronic health conditions	
Hypertension	141 (54.4)
Diabetes	57 (22.0)
HIV/AIDS	7 (2.7)
Mental health condition	146 (56.4)
Tobacco use	126 (48.6)
Substance use disorder	89 (34.4)
Total days at hotel	
< 7 d	18 (7.0)
7-30 d	77 (29.7)
31-90 d	63 (24.3)
>90 d	101 (39.0)
SARS-CoV-2/COVID-19 outcomes	
Positive test, overall	69 (26.6)
Positive test, protective housing^b^	11 (4.2)
Hospitalization	11 (4.2)
Death	0

^a^ Other race and ethnicity categories included non-Hispanic Asian and uncategorized.

^b^ Of the 69 total positive tests at the hotel, 11 were ascertained from protective housing participants (n = 201).

### SARS-CoV-2 Infection and COVID-19 Outcomes

In total, 201 PEH were included in the protective housing cohort, of which 11 tested positive for SARS-CoV-2 during the study period. Seven of the 11 positive tests (63.6%) were obtained within 5 days of admission to the hotel. The overall incidence of COVID-19 among protective housing participants was 54.7 per 1000 people (95% CI, 22.4-87.1 per 1000) compared with 137.1 per 1000 (95% CI, 125.1-149.1 per 1000) among PEH residing in shelters citywide (*P* = .001). Monthly analysis found a large difference between cohorts in April 2020, when the bulk of outbreaks were occurring in congregate shelters, followed by a decline in incidence to low levels across both cohorts (Figure).

**Figure.**
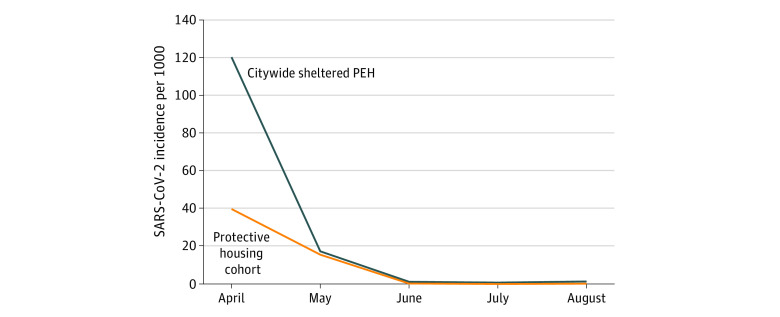
Monthly Incidence of SARS-CoV-2 Infection Among Intervention Participants Compared With Citywide People Experiencing Homelessness (PEH), Chicago, Illinois, 2020

Among all 259 PEH admitted to the hotel, 11 (4.2%) were transferred to a hospital for severe illness due to COVID-19 (eTable 1 in the Supplement), but none died; 2 belonged to the protective housing cohort.

### Chronic Health Conditions

Among all study participants (N = 259), 141 (54.4%) had a self-reported underlying diagnosis of hypertension, of which 84 had a measured SBP greater than or equal to 130 mm Hg or DBP greater than or equal to 80 mm Hg^17^; 57 (22.0%) had a self-reported underlying diagnosis of diabetes, of which 23 had a measured HbA_1c_ greater than 7%.^18^ In models examining change in chronic illness measurements during the intervention period relative to baseline (Table 2), we observed an adjusted change in SBP of −5.7 mm Hg (95% CI, −9.3 to −2.1) and HbA_1c_ of −1.4% (95% CI, −2.4% to −0.4%). We observed no significant changes in DBP during the intervention period. In sensitivity analyses using fixed effects models, changes were substantively similar (eTable 2 in the Supplement).

**Table 2.** Population Average Change in Blood Pressure and Glycemic Control Relative to Baseline, Chicago, Illinois, 2020

Health measurement	Baseline, mean (SD)	Population average change (95% CI)^a^	*P* value
Systolic blood pressure, mm Hg^b^	139.6 (20.8)	−5.7 (−9.3 to −2.1)	.002
Diastolic blood pressure, mm Hg^b^	83.0 (12.9)	0.6 (−2.7 to 4.0)	.72
Hemoglobin A_1c_, %^c^	8.3 (2.6)	−1.4 (−2.4 to −0.4)	.006

Abbreviation: SUDs, substance use disorders.

^a^ Estimates derived from generalized estimating equations (GEE) in population-averaged longitudinal regression models including a random effect for patient and robust standard errors; models adjusted for sociodemographic characteristics (age, gender, race and ethnicity, and preferred language), comorbid mental health conditions and/or SUDs, shelter of origin, day at the hotel (ie, when the observation was recorded), and total days at the hotel.

^b^ Analytic sample included 1216 blood pressure measurements from 136 participants.

^c^ Analytic sample included 63 A_1c_ measurements from 41 participants; HbA_1c_ measurements obtained within 2 weeks of initial measurements were excluded due to limited change within a short time interval.

### Mental Health Conditions and Substance Use Disorders

Among all 259 study participants, 146 (56.4%) had mental health conditions (eTable 3 in the Supplement). Of those with mental health conditions, 80 (54.8%) were taking medication(s) on admission and 41 (28.1%) were initiated on medications during admission. The majority of these participants (n = 126; 86.3%) received at least one behavioral health encounter, and more than half (n = 91; 62.3%) reported that their mental health improved or stabilized during their stay.

Eighty-nine participants (34.4%) had SUD, and 52 (20.1%) had both mental health and SUD diagnoses. Of those with SUD, 24 were supplied daily methadone or sublingual buprenorphine-naloxone, and 9 were newly initiated on buprenorphine-naloxone. Nearly half of participants (n = 126, 48.6%) used tobacco, of which 80 received medication to reduce or stop tobacco use.

### Housing and Other Social Services

Approximately half of all study participants (n = 132, 51.0%) departed the hotel to some form of permanent, transitional, or other housing (Table 3). Of the remaining 127, a small proportion (n = 26) chose to leave the hotel on their own to an unknown destination. A large proportion (n = 83) were transferred to an isolation facility, hospital, or residential recovery program to receive treatment for medical illnesses, psychiatric conditions, and/or SUDs. Other social services included assistance with obtaining voter’s registration cards (n = 27), other identification cards (n = 27), CARES Act stimulus checks (n = 26), Medicaid (n = 21), and transportation disability cards (n = 11).

**Table 3.** Participant Destinations Upon Hotel Departure

Destination	Participants, No. (%) (N = 259)
Housing	
Permanent housing programs	
Rapid Rehousing	63 (24.3)
Permanent supportive housing	32 (12.4)
Chicago Housing Authority	7 (2.7)
Front door diversion^a^	4 (1.5)
Transitional/bridge housing	8 (3.1)
Other housing	
Self-arranged housing	12 (4.6)
Specialized Mental Health Recovery Facility	6 (2.3)
Medical facility	
COVID-19 isolation facility	39 (15.1)
Hospital facility	20 (7.7)
Residential recovery program	24 (9.3)
Voluntary return to shelter	18 (6.9)
Voluntary departure to unknown destination	26 (10.0)

^a^ Front Door Diversion is a program provided by the Illinois Department of Human Services to Medicaid-eligible persons who may otherwise require admission to a nursing-level care facility; services support maintenance of mental health conditions in a community-based setting.

## Discussion

In this Chicago-based cohort study of persons experiencing homelessness who were at increased risk of severe illness from COVID-19, we found that a rapid-entry hotel-based protective housing intervention was associated with a 2.5-fold reduction in the incidence of SARS-CoV-2 infection compared with citywide rates among sheltered PEH. These findings suggest that targeting high-risk individuals for protective supportive housing can be an effective strategy to minimize morbidity and mortality due to COVID-19. Protective housing, in addition to quarantine and isolation facilities, will be key to building resiliency against the potential for ongoing surges of COVID-19, as well as other local disease outbreaks.

Beyond emergency preparedness, our study has several implications for policies regarding the intersecting health and social needs of PEH. First, in addition to improvements in COVID-19 outcomes, we documented improvements in disease control, as well as support for participants’ mental health and SUD. We observed a reduction in SBP of 5.7 mm Hg, consistent with a moderate effect in intervention-based studies.^18,19^ We observed similarly moderate reductions in HbA_1c_ compared with meta-analyses of lifestyle change^19^ and drug therapy.^20^ In addition, 28.1% of participants with a mental health condition were newly initiated on psychiatric medications; and 10.1% of participants with SUD were newly initiated on treatment for opiate use disorder. We speculate that temporary stabilization housing for PEH with complex medical, behavioral, and social needs may be an important means of interrupting cyclical utilization of emergency department and hospital services, as well as cycling through jails, prisons, and other settings.^21^

Second, the intervention addressed gaps in Chicago’s shelter to housing continuum. The pandemic presented a unique opportunity for multisector coordination between city agencies, shelters, primary care clinics, behavioral health and SUD specialists, and housing organizations, resulting in more streamlined case management and housing placements. From a policy standpoint, COVID-19–related infusion of emergency response funding and resources may provide city public health departments with an unprecedented opportunity to explore noncongregate shelter options with varying levels of medical supports that will better serve residents in need of temporary housing. The City of Chicago, for example, broadly recognized the importance of transforming the physical footprint of its emergency housing system to reduce reliance on large congregate facilities, particularly for individuals at high risk of adverse outcomes in those settings. Importantly, flexible, integrated, and sustained funding sources at the federal, state, and local levels are needed to enable acquisition, renovation, and capital improvement of new and existing shelter facilities. Support is also needed to fund initiatives that proactively identify PEH with high-risk medical and behavioral health needs, and provide intensive, whole-person services to stabilize their conditions and facilitate a successful transition to permanent supportive housing.

### Limitations

This study has several limitations. First, measurement of SARS-CoV-2 infections among citywide PEH was based on reporting to CDPH. Although enhanced reporting (eMethods in the Supplement) sought to maximize reporting among PEH, it is likely that measurement was still incomplete, resulting in an underestimate of the citywide incidence. Conversely, the incidence of SARS-CoV-2 in the intervention cohort was likely overestimated, because a large proportion of positive tests (n = 7, 63.6%) were obtained within 5 days of admission to the hotel, making asymptomatic infection prior to arrival highly probable. These limitations are likely to bias results toward the null hypothesis and underestimate the reported association.

Second, intervention participants were comprised of a voluntary sample from Chicago-based shelters and recruited under real-world conditions during a public health emergency, making selection bias highly likely. Recruitment targeted PEH at high risk for severe illness due to COVID-19. As such, intervention participants were older and had substantial medical and psychiatric comorbidities relative to citywide PEH. It is also notable that staff members went to several encampments to discuss participation with unsheltered PEH, and none of these individuals agreed to participate.

Third, this is a single-site study of a novel protective housing model for PEH in Chicago and may not be widely generalizable. However, this intervention is likely replicable in other dense, urban areas where congregate settings are a predominant form of housing for PEH. Fourth, social distancing and infection control measures made medical interventions more challenging, which potentially limited improvements in the management of chronic conditions. Fifth, because a comparison group was not available for secondary measures (BP and HbA_1c_), it is possible that observed reductions may be due to regression to the mean.

Finally, in response to George Floyd’s murder and resulting demonstrations that occurred directly outside the hotel, the intervention team eased restrictions for movement to and from the hotel, thereby increasing participant autonomy. Although this increased movement may have impacted the outcome of the protective housing intervention, these steps were necessary to recognize and address the role of systemic racism in perpetuating health and housing inequities among PEH.

## Conclusions

This cohort study found that a hotel-based protective housing intervention was associated with marked reduction in the incidence of SARS-CoV-2 infection among study participants. A large proportion of PEH who received integrated medical and social services also appeared to achieve improvements in their chronic health conditions, received treatment for mental health conditions and SUDs, and departed the intervention to permanent housing. This model is not only relevant for the pandemic era, but is a critical piece to addressing the heterogeneous needs of PEH across the US.

## Notes

Supplement. **eMethods.** Estimating Citywide Incidence of SARS-CoV-2 Infection among Persons Experiencing Homelessness (PEH) **eFigure.** Intervention Participation by Shelter Origin, Chicago, IL, 2020 **eTable 1.** Detailed COVID-19 Severe Illness Outcomes, Chicago, IL, 2020 **eTable 2.** Participant Change in Blood Pressure and Glycemic Control Relative to Baseline, Chicago, IL, 2020 **eTable 3.** Detailed Mental Health and Substance Use Diagnoses, Chicago, IL, 2020
