# Supplementary material for: Assessment of a Hotel-Based Protective Housing Program for Incidence of SARS-CoV-2 Infection and Management of Chronic Illness Among Persons Experiencing Homelessness
Source: JAMA Netw Open. 2021 Dec 13;4(12):e2138464. doi: 10.1001/jamanetworkopen.2021.38464 (PMC8669521; doi:10.1001/jamanetworkopen.2021.38464)
Supplement: Supplement. — eMethods. Estimating Citywide Incidence of SARS-CoV-2 Infection among Persons Experiencing Homelessness (PEH) eFigure. Intervention Participation by Shelter Origin, Chicago, IL, 2020 eTable 1. Detailed COVID-19 Severe Illness Outcomes, Chicago, IL, 2020 eTable 2. Participant Change in Blood Pressure and Glycemic Control Relative to Baseline, Chicago, IL, 2020 eTable 3. Detailed Mental Health and Substance Use Diagnoses, Chicago, IL, 2020 [file jamanetwopen-e2138464-s001.pdf]

Supplemental Online Content

Huggett TD, Tung EL, Cunningham M, et al. Assessment of a hotel-based protective housing program for incidence of SARS-CoV-2 infection and management of chronic illness among persons experiencing homelessness. *JAMA Netw Open.* 2021;4(12):e2138464. doi:10.1001/jamanetworkopen.2021.38464

eMethods. Estimating Citywide Incidence of SARS-CoV-2 Infection among Persons Experiencing Homelessness (PEH)

eFigure. Intervention Participation by Shelter Origin, Chicago, IL, 2020

eTable 1. Detailed COVID-19 Severe Illness Outcomes, Chicago, IL, 2020

eTable 2. Participant Change in Blood Pressure and Glycemic Control Relative to Baseline, Chicago, IL, 2020

eTable 3. Detailed Mental Health and Substance Use Diagnoses,

Chicago, IL, 2020

This supplemental material has been provided by the authors to give readers additional information about their work.

eMethods. Estimating Citywide Incidence of SARS-CoV-2 Infection among Persons Experiencing Homelessness (PEH)

SARS-CoV-2 infections among PEH in Chicago were ascertained through enhanced reporting to the Chicago Department of Public Health (CDPH). It is mandatory to report all cases of SARS-CoV-2 infection in Chicago through the Illinois' National Electronic Disease Surveillance System (I-NEDSS). Cases reported to I-NEDSS with an address that matched that of a known homeless shelter were flagged for direct outreach to shelter service providers. As of March 19, 2020, all congregate living facilities became additionally required to report to CDPH if ≥2 cases were identified among residents or staff at their facility. Homeless service providers were encouraged to report individual cases via a widely publicized online reporting form and CDPH staff conducted regular telephone outreach to shelter service providers to inquire about unreported or unmatched cases. If a case in a resident or staff member at a homeless shelter was identified, teams of clinicians contracted by CDPH conducted widespread RT-PCR testing of all consenting residents at the address of the index case, regardless of symptom or reported exposure status. These results were provided directly to CDPH, in addition to the mandatory reporting outlined above. Case detection methods are described in more detail in: https://academic.oup.com/ofid/article/7/11/ofaa477/5921124

For this paper, incidence was defined as the number of SARS-CoV-2 infections among PEH residing in homeless shelters with symptom onset or specimen collection date (whichever was earlier) between April 2 and September 3, 2020, divided by the total susceptible population of sheltered PEH in Chicago. Incidence rates were calculated for each month of the intervention, as well as for the full study period. Of note, PEH living in encampments or on the street were excluded from this analysis, as no study participants were recruited from these settings. The total sheltered population (n=3,861) was based on the most-recent Point-In-Time count, which was led by the Chicago Department of Family and Support Services and conducted in January 2020 following approved methodology from the United States Department of Housing and Urban Development. This methodology can be found at: https://www.chicago.gov/content/dam/city/depts/fss/supp_info/Homeless/2020/2020%20PIT%20Report_vFinal.pdf. From this total, individuals that were admitted to the hotel as part of the protective housing cohort (n=204) were excluded for the initial denominator, as well as any sheltered PEH who were positive for COVID-19 prior to April 2. The denominator was subsequently adjusted monthly to further remove any sheltered PEH known to have been infected during the previous month.

© 2021 Huggett TD et al. *JAMA Network Open.*

eFigure. Intervention Participation by Shelter Origin, Chicago IL, 2020

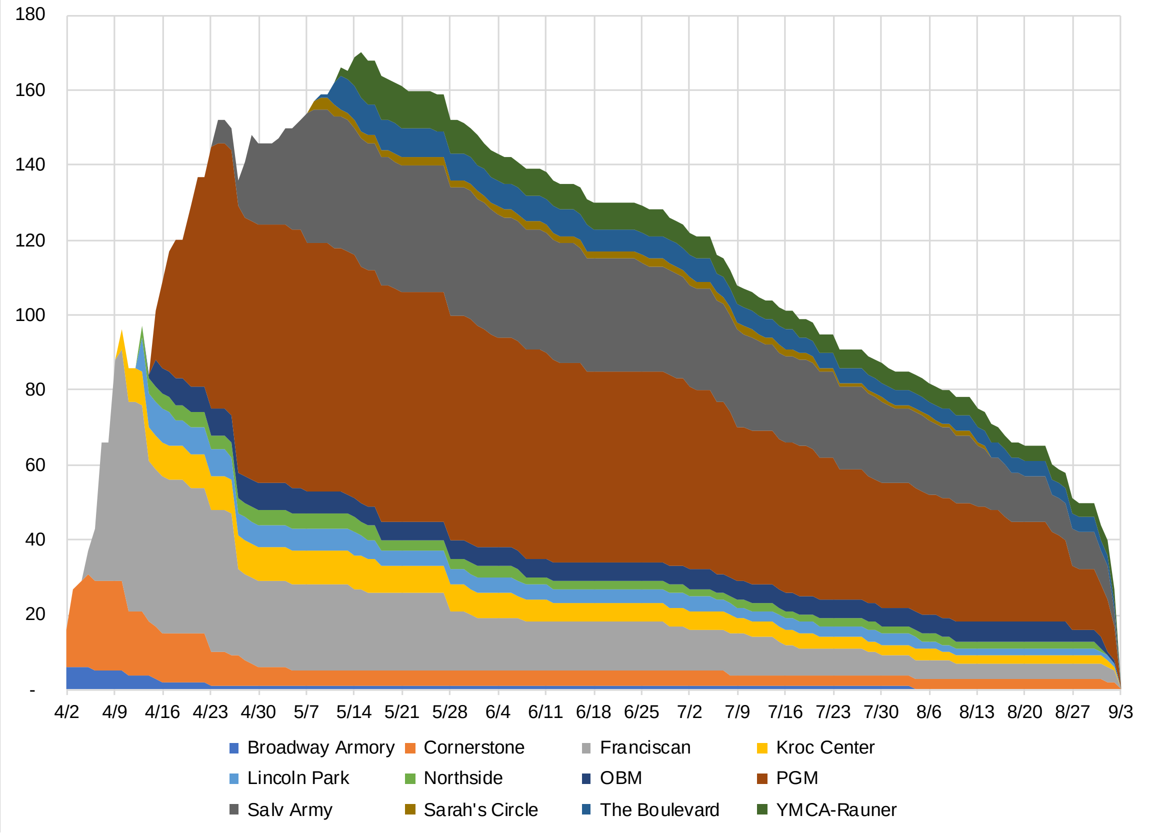

Figure shows participation in the intervention over time by shelter of origin; the peak census (n=172) occurred between May 16-18, 2020.

© 2021 Huggett TD et al. *JAMA Network Open.*

eTable 1. Detailed COVID-19 Severe Illness Outcomes, Chicago, IL 2020

**COVID-19 Severe Illness Outcomes** N=11	n
Hospitalization[a]	11
Intensive care unit admission	8
Intubation and mechanical ventilation	6
Death	0
Primary reason for hospital referral[a]	
Hypoxia (%O2 <90%)	7
Altered mental status	1
Bradycardia	1
Chest pain	1
Hypoglycemia	1
Psychiatric decompensation	1

[a]In total, 11 intervention participants were hospitalized due to severe COVID-19, with 1 participant hospitalized twice; 2 were from the protective housing cohort.

© 2021 Huggett TD et al. *JAMA Network Open.*

eTable 2. Participant Change in Blood Pressure and Glycemic Control Relative to Baseline, Chicago IL, 2020

**Health Measurement**	**Baseline Mean** (Std. Dev.)	**Within-Subject Change** (95% CI)[a]	*P*-value
Systolic Blood Pressure[b] (mmHg)	139.6 (20.8)	-6.6 (-10.3, -2.9)	0.001
Diastolic Blood Pressure[b] (mmHg)	83.0 (12.9)	-1.3 (-4.4, 1.7)	0.38
Hemoglobin A1c[c] (%)	8.3 (2.6)	-1.9 (-3.2, -0.7)	0.004

[a]Estimates derived from fixed effects longitudinal panel models including robust standard errors; models accounted for day at the hotel (i.e., when the observation was recorded).
[b]Analytic sample included 1,216 blood pressure measurements from 136 participants.
[c]Analytic sample included 63 A1c measurements from 41 participants.

© 2021 Huggett TD et al. *JAMA Network Open.*

eTable 3. Detailed Mental Health and Substance Use Diagnoses, Chicago, IL 2020

**Participant Diagnoses** N=259	n	%
Mental health condition	146	56.4
Substance use disorder	89	34.4
Both mental health condition and substance use disorder	52	20.1
**Detailed Diagnosis**	n	%
Mental health diagnosis (N=146)		
Depression	33	22.6
Schizophrenia/schizoaffective	24	16.4
Adjustment disorder	21	14.4
Bipolar disorder	19	13.0
Anxiety	15	10.3
Post-traumatic stress disorder	15	10.3
Insomnia	13	8.9
Psychotic disorder NOS	5	3.4
Bereavement	5	3.4
Delusional disorder	4	2.7
Personality disorder	3	2.1
Obsessive compulsive disorder	1	0.7
Eating disorder	1	0.7
Substance use disorder diagnosis (N=89)		
Opiate use disorder	35	39.3
Alcohol use disorder	34	38.2
Cocaine use	29	32.6
Other	12	13.5

© 2021 Huggett TD et al. *JAMA Network Open.*
